# Management of LC Type I (LC-1) Pelvic Injuries with Complete Sacral Fracture: Comparison of Solitary Anterior Fixation with Combined Anterior-Posterior Fixation

**DOI:** 10.1155/2022/3918794

**Published:** 2022-01-18

**Authors:** Pengfei Wang, Syed Haider Ali, Chen Fei, Binfei Zhang, Xing Wei, Hu Wang, Yuxuan Cong, Hongli Deng, Yahui Fu, Kun Zhang, Yan Zhuang

**Affiliations:** ^1^Department of Orthopedics and Traumatology, Xi'an Honghui Hospital, Xi'an Jiao Tong University Health Science Center, Xi'an, No. 555, East Friendship Road, 710054 Xi'an City, Shaanxi Province, China; ^2^Institute for Global Orthopaedics & Traumatology, University of California, 2550 23rd St., Bldg.9, 2nd Floor San Francisco, CA, 94110 San Francisco, USA

## Abstract

**Background:**

Management of LC-1 type pelvic injuries, particularly in patients with complete sacral fracture (LC-1 PICSF, OTA type 61-B2.1), remains controversial. Specific indications for solitary fixation remain unclear, and there is a paucity of outcomes data in comparison to combined fixation. We undertook a retrospective study in patients with LC-1 PICSFs to compare outcomes between solitary anterior fixation and combined anterior-posterior fixation.

**Methods:**

A retrospective cohort study was conducted with enrollment from 2016 to 2018 at a single tertiary-referral center in China. Adults with operatively managed LC-1 PICSFs were enrolled. Patients with sacral displacement < 1 cm as assessed by axial CT received solitary anterior ring fixation (group A); patients with displacement ≥ 1 cm received combined fixation of both the anterior and posterior rings (group B). Reduction was confirmed by manipulation under anesthesia. Patients followed up for at least 24 months postoperatively. Primary outcome was function (Majeed score). Secondary outcomes included intraoperative characteristics, pain (VAS score), quality of fracture reduction (Tornetta and Matta radiographic grading), rate of nonunion, early weight-bearing status, and complication rate.

**Results:**

68 (89%) of 76 enrolled patients completed follow-up. Patients in group A exhibited improved operative times, less time under fluoroscopy, and less blood loss as compared to group B. There were no significant differences between groups A and B regarding quality of fracture reduction, rate of union, functional outcomes, or rate of complications. Notably, group B patients were more likely to achieve full early weight-bearing.

**Conclusion:**

LC-1 PFCSFs can get benefits from ORIF; the treatment algorithm should be differently made following the degree of the sacral fractures displacement. Less than 1 cm sacral fracture displacement may get good functional outcomes from solitary anterior fixation. However, for the sacral fractures displacement greater or equal to 1 cm, both the anterior and posterior pelvic rings should be surgical stabilization.

## 1. Introduction

Young and Burgess lateral compression type I (LC-1) fractures account for up to 63% of all pelvic ring injuries, but optimal treatment remains controversial [1–6]. This fracture pattern is usually caused by a lateral impact force and is characterized by pubic ramus fractures and sacral compression fractures without vertical instability [2, 3]. Although the incidence of LC-1 pelvic fractures is high, the optimal treatment algorithm remains under debate [2, 4–6]. The assessment of stability in LC-1 pelvic ring fractures is an important factor in the treatment algorithm. Some authors [5–7] advocate a nonoperative treatment based on the original work by Young and Burgess which views this fracture pattern as a stable injury because the main ligaments contributing to pelvic stability remain intact [2]. Others dispute the increased rate of complications in the surgically managed patient compared to those receiving nonoperative treatment [8–10].

Attempts have been made to qualify pelvic ring stability in LC-1 type fracture patterns by means of radiographic assessment [6]. Others advocate the notion of not relying merely on low-sensitivity plain radiographs to evaluate stability [8, 11, 12]. One of the reasons for this is because based on CT scans LC-1 injuries represent a spectrum of injury severity, different fracture anatomy, and potential instability [13]. Furthermore, assessment of pelvic ring stability through manipulation under anesthesia (MUA) revealed that almost 40% of LC-1 injuries were inherently unstable [14]. A recent prospective study utilizing intraoperative MUA to assess stability found that LC-1 injuries with a complete posterior sacral injury were all inheritably rotationally unstable and patients presenting with these fracture patterns would gain from surgical stabilization [15]. This has been confirmed by Bruce et al. that reported that LC-1 fractures with complete sacral fractures and bilateral rami fractures displace at a significantly greater rate [16].

In case of a surgical intervention, most surgeons would likely fix the posterior pelvic ring fractures with a cannulated screw followed by the anterior pelvic ring fixation. Difficulties in reducing and fixating the posterior ring may arise in cases where the anterior ring is significantly displaced. The necessity of perfect posterior ring reduction and fixation is debatable since there is evidence that an anterior internal fixator can provide some indirect compression along the sacroiliac joint, which is beneficial and possibly sufficient for the stability of the pelvic ring [17]. To the best of our knowledge, there has been no study comparing the long-term functional outcomes and complications between surgically managed LC-1 pelvic fractures with different degrees of posterior ring displacement. The hypothesis is that the treatment algorithm of LC-1 PFCSFs should be separately made according to the degree of the sacral fractures displacement. Minimal sacral fracture displacement may get good functional outcomes from solitary anterior fixation; nevertheless, greater sacral fracture displacement should surgically stabilize both the anterior and posterior pelvic rings.

## 2. Patients and Methods

This retrospective comparative study was approved by our Institutional Review Board (No. 201604006). All patients who were admitted to our institution and fulfilled the following criteria were enrolled in the study. Inclusion criteria were (a) LC-1 pelvic fracture (OTA 61-B2.1) with a complete sacral fracture. This was defined as a visible fracture through the anterior and posterior cortices of the sacrum at the S1 level on the axial CT (computerized tomography) scan. (b) Patients older than 18 years, (c) have no signs of a pathological fracture, and (d) complete follow-up available. Patients with incomplete sacral fractures or those with an associated acetabular fracture were excluded.

All the patients were evaluated preoperatively using a standard radiological protocol including AP view, inlet view, outlet view, and pelvic CT scan with three-dimensional reconstructions. The Young and Burgess classification systems was used to classify the fracture patterns. The axial CT scans at the S1 level were used for the measurement of fracture displacement of the posterior ring. A sacral fracture was deemed complete if a fracture line was visible through both the anterior and posterior cortices. The distance between either the anterior cortices or posterior cortices was measured at the S1 level.

Patients were allocated into one of two groups depending on the amount of displacement of the sacral fractures. Group 1 was comprised of patients with LC1 fractures with a displacement of less than 1 cm. Group 2 was comprised by those that sustained LC-1 fractures with a displacement greater or equal to 1 cm.

The allocation and the treatment plan was made by two senior pelvic surgeons(Y Z and K Z). In case of controversy, a consensus is achieved by the majority of senior experienced surgeons in a morning review meeting. In group 1, only the anterior pelvic fractures were reduced and fixed using plate, cannulated screws, infix, external fixator depending on the fracture patterns and local soft tissue condition. Patients in group 2 underwent anterior pelvic fracture fixation in combination with sacral fracture fixation.

After successful induction of general anesthesia, the patient was placed supine on a radiolucent table. In group 1, the anterior pelvic fracture was reduced and fixed through a Stoppa approach or ilioinguinal approach. In some cases, closed reduction and 6.5 mm diameter cannulated screw, or the infix [18] or exfix was inserted percutaneously. To avoid joint and intrapelvic penetration as well as for confirmation of screw positioning. the procedure was performed under fluoroscopic control. Once the anterior ring fixation was completed the reduction and stability of the pelvis were examined and confirmed under the anesthesia with the surveillance of the fluoroscopy. The examination consisted of a resting static film followed by internal rotation, external rotation, and push–pull maneuvers of both lower extremities. Each of these maneuvers was performed using the anteroposterior, inlet, and outlet projections [14]. In group 2, the techniques for reduction and fixation of anterior pelvic fractures were identical to those used in group 1 The posterior pelvic fractures were fixed by percutaneous sacroiliac joint screws. For dysmorphic sacral osseous pathways, techniques used were the tension band plate or pedicle screws connected to a transverse rod [19].

### 2.1. Postoperative Management

Prophylactic use of antibiotics (cefazolin, 1.0 g, three times a day, LuKang pharmaceutical Co., China) was continued for 24-48 hours postoperatively. Intermittent compression devices and low molecular weight heparin (LMWH, 4100 U, once a day, GlaxoSmithKline Co., UK) were used for venous thromboembolism (VTE) prophylaxis during hospital stay. After discharge, patients used 10 mg of rivaroxaban once a day until five weeks after the surgery. The postoperative rehabilitation protocol consisted of protected toe-touch weight-bearing for 6 weeks in group 1 and immediate full weight-bearing activity in Group 2. Patients who are not able to safely ambulate were allowed to bed-to-chair transfers with assistance until they are advanced to partial weight-bearing activity at 4-6 weeks.

### 2.2. Data Collection

The patients were followed up by an independent senior pelvic surgeon (Xing Wei) who were not involved in the surgery or the definitive care.

Baseline characteristics, operative data, and post-operative data that were collected were as follows: (1) demographics: gender, age, and mechanism of the accident; (2) fracture type according to the Young and Burgess classification, injury pattern of the anterior and posterior pelvic ring; (3) injury severity score (ISS); (4) visual analog score (VAS, pre- and postoperative at 72 hours), and (5) surgery-related variables: time of operation, blood loss, fluoroscopy time, and quality of the reduction according to the Tornetta and Matta radiographic grading [20]; (6) early weight-bearing status; and (7) functional outcome according to the most often used Majeed score grading [21]. Finally, all the preoperative or postoperative complications were recorded.

Follow-up evaluation included postoperative visits at 1, 2, 3, and 6 months, one year, and annually thereafter. The Majeed score grading was used to assess the clinical and functional outcome with regard to pain (30 points), standing (36 points), sitting (10 points), sexual (4 points) function, and walking (20 points). The total score was graded as excellent (≥85 points), good (70–84 points), fair (55–69 points), or poor (<55 points). The Tornetta and Matta radiographic grading assesses reduction and classifies displacement of less than 5 mm as excellent, 5-10 mm as good, 11–20 mm as fair, and >20 mm as poor.

### 2.3. Statistics

IBM SPSS 19 statistical software (Chicago, IL, USA) was used for statistical analysis. Data were presented as means and standard deviations. Chi-square and Fisher exact tests were performed for the categorical variables as appropriate. Student's *t*-test was used for comparison of continuous variables. A *p* value of 0.05 was defined as statistically significant.

## 3. Results

Between June 2016 to December 2018, 180 pelvic ring fractures were treated at our level 1 trauma institution. Sixty-eight patients (37.8%) met all the inclusion criteria and were included in this study. There were 37 males and 31 females with a mean age of 39.4 years (range, 18-71) included. All patients were followed for an average of 29.3 ± 5.9 months (range, 24-42 months). All fractures proceeded to union. The demographic data, trauma mechanism, additional injuries, ISS, and anterior ring injury patterns are presented in Table 1. A significant difference in trauma mechanism between the two groups (*p* < 0.05), was identified but all other baseline characteristics were comparable.

**Figure 1:**
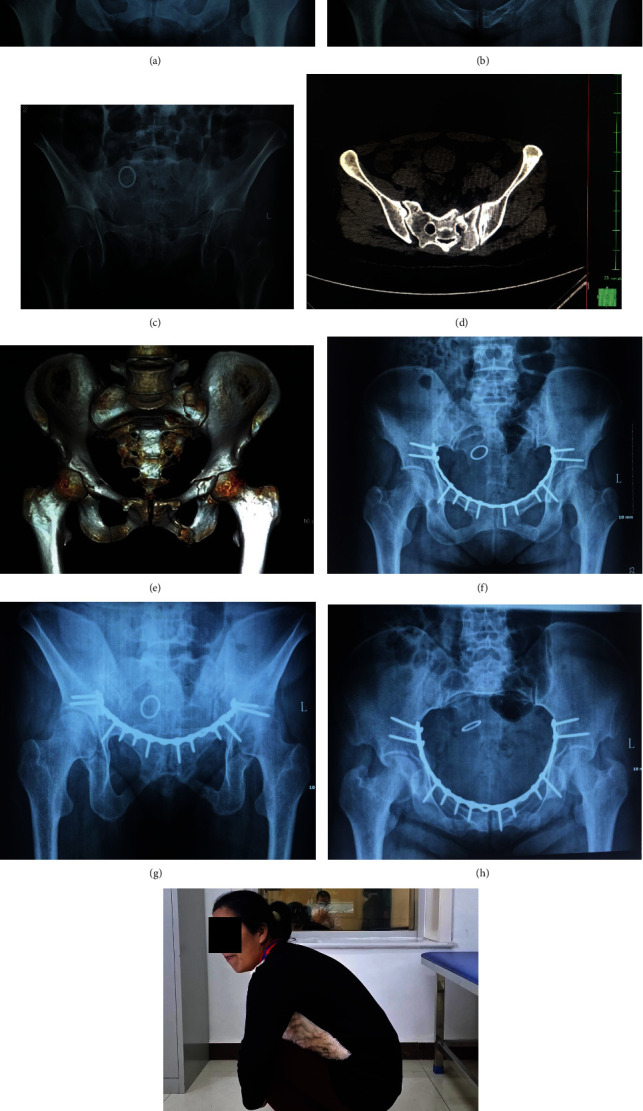
A 43-year-old female presenting with pelvic ring fractures (Young and Burgess LC-1/Tile B2). Preoperative AP view (a), inlet view(b), and outlet view(c) showed both pubic rami and a transforaminal sacral fracture on the left side. The CT scan (d) and 3D-reconstruction (e) showed the sagittal fracture line across the anterior and posterior cortex of S2 with minimal displacement. The pubic rami fractures were fixed through modified Stoppa approach. At final follow-up, the radiographs (f–h) and functional photographs (i) showed that the fractures healed, and the function recovered well.

**Figure 2:**
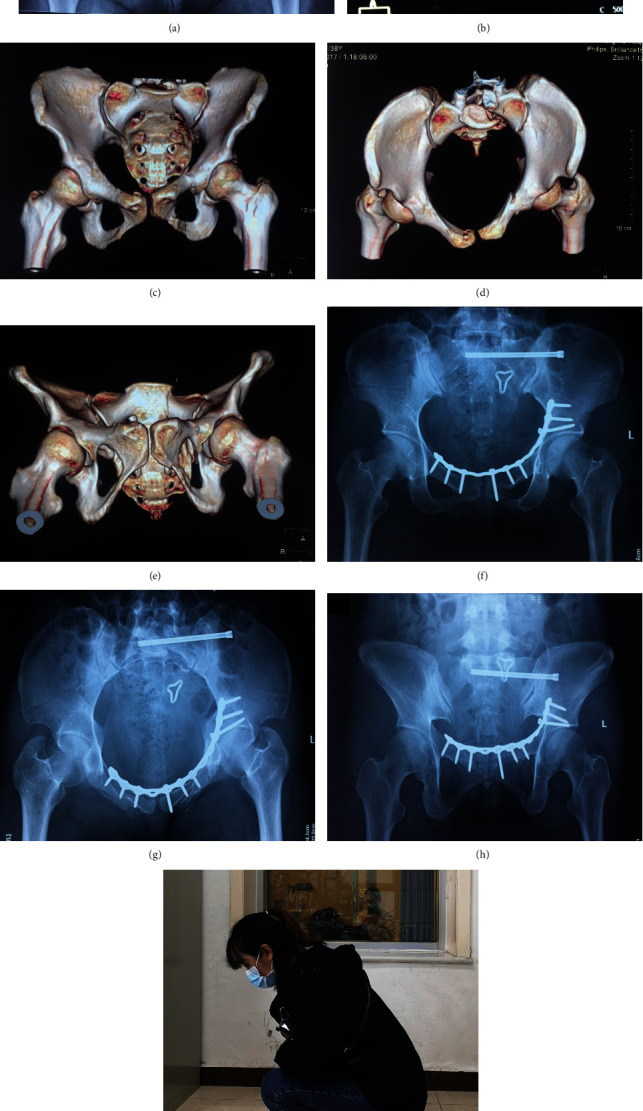
Radiographs of 32-year-old female with pelvic ring fractures (Young and Burgess LC-1/Tile B2). Preoperative AP view (a), CT scan (b), and 3-D reconstruction (c–e) showed left pubic rami and ipsilateral transforaminal sacral fracture with significant displacement. The pubic rami fracture was reduced and fixed through modified Stoppa approach followed by a sacroiliac screw inserted percutaneously. Postoperative radiographs (f–h) showed the reduction and fixation was well. The functional photograph (i) showed recovered well.

After anterior fixation of the pelvic ring fractures in group 1, all were deemed stable after MUA. At the final follow-up, reduction of the sacral fractures according to the Tornetta and Matta radiographic grading was excellent and good for group 1 (91.7%) and group 2 (87.6%), respectively. The functional outcome score according to Majeed scores grading was excellent in both groups (88.9% and 87.5% in Group 1 and Group 2, respectively.) (Figures 1 and 2). There were no statistically significant differences in the Tornetta and Matta radiographic grading and Majeed grading scores between group 1 and group 2 (*p* > 0.05). The mean operation time, the mean blood loss, and the mean radiological time in group 1 were significantly less than in group 2, respectively (117.6 ± 46.0 vs. 158.9 ± 28.1 min, 187.5 ± 133.0 vs. 264.1 ± 158.2 ml, and 12.58 ± 3.7 vs. 74.28 ± 18.8 second; *p* < 0.05). There was a significant difference in weight-bearing status between these two groups (*p* < 0.05) (Table 2).

Fourteen patients developed venous thromboembolism (VTE). 13 patients were diagnosed with deep vein thrombosis (DVT) including 6 DVT in group1 and 7 DVT and 1 nonfatal pulmonary embolism (PE) in group 2. There were no deep infections noted. Four patients were diagnosed with L5 nerve root neurological deficits. In group 1, one patient preoperatively presented paresthesia located in the dorsal of the foot. The other 3 patients were in group 2. Two of these three patients present preoperatively, the other one was injured during the surgery. All the patients with L5 nerve root neurological deficits completely recovered except for the patient who had the iatrogenic L5 injury in group 2. The patient suffered from residual weakness of the extensor hallucis longus. Surgical exploration and decompression were offered, but the patient refused. Three patients had implant-related complications. Two patients were in group 1 and one patient was in group 2, respectively. These three patients included one patient with a pubic rim delayed union and loosening of screws in the anterior plate without symptoms. One patient experienced injury to the lateral femoral cutaneous nerve (LFCN) presenting as paresthesia. The symptom of LFCN recovered fully after implant removed at six months. The other patient experienced hardware irritation caused by a pedicle screw. After removal of hardware at three months, the symptoms dissipated. No heterotopic ossifications or traumatic sacroiliac arthritis was observed. There was no significant difference in complication rates between these two groups (*p* > 0.05) (Table 3).

## 4. Discussion

The current study found that LC-1 PFCSFs could get benefits from the open reduction and internal fixation. The treatment algorithm should be separately made according to the degree of the sacral fractures displacement.

Stable LC-1 fractures can get good outcomes from conservative treatment because they will not displace under normal physiological weight bearing. Conversely, unstable patterns or displaced fractures are usually treated with surgical reduction and stabilization [22–24].

Determining whether or not an LC-1 fracture is stable remains challenging [9]. Magnetic resonance imaging and ultrasonography have been proposed as adjuncts, but their clinical utility has not yet been elucidated [25, 26]. Recently, the predicted value of pelvic ring instability by positive MUA was verified by several studies [14, 15]. By intraoperative MUA, Tosounidis et al. [15] demonstrated that the LC-1 injuries with a complete posterior sacral injury are inheritably rotationally unstable. On the other hand, Whiting et al. reported that immediate weight-bearing as tolerated seems safe in patients with pelvic ring injuries who have had a negative MUA [7]. Although the MUA may be the most reliable, it requires general anesthesia before make surgical treatment decision, which may not be cost-effective. For evaluation of pelvic ring stability in LC-1 type fractures, the X-ray or CT scan is widely used. According to Bruce et al. using CT scans, complete sacral fracture and bilateral rami fractures displace more prone to future displacement [16].

Previous clinical and biomechanical study [17, 27, 28] found that in partially unstable LC-1 fractures, fixation of the anterior pelvic ring only, which was similar to Group 1 in the current study, can provide some indirect compression along the sacroiliac joints, which can be beneficial for the functional outcomes. We compare the outcomes between the solitary anterior fixation and anterior-posterior pelvic ring fixation. Kanakaris et al. [28] carried out a comparative study between the displacement of anterior or posterior pelvic fractures less 5 mm and more than 2 cm assessment during the examination with fluoroscopy under anesthesia (EUA). However, the spectrum was broad; they did not mention the intermediate displacement of pelvic fractures. Although Gaski et al. [5] investigated the intermediate displacement which was limited to the initial displacement less than 1 cm by the measurement in the plain radiographs, however, the measurement has the inherently inaccurate defect, which has been confirmed by the previous study. Lin et al. reported that 45.5% of patients with bilateral ramus fractures and 42.0% of patients with dual-ramus fractures had concomitant sacral fractures not observed on plain radiographs yet [11], let alone the accurate measurement. This issue can be solved by the CT scan. In the current study, we used 1 cm measured in the CT scan as the threshold to divide the displacement was much more reasonable.

Surgical intervention in these two groups was associated with significantly reduced pain postoperatively (3.05 ± 0.98 in group 1 and 4.06 ± 1.32 in group 2). The results were similar to the previous reports [15, 19]. These results support the view that surgical intervention provides significant pain relief and allow early ambulation. Simultaneously, in solitary anterior fixation (group 1), the operation time was reduced significantly (117.6 ± 46.0 vs. 158.9 ± 28.1 mins), the saved time was similar to the previous reports [29, 30]. With the shortening of the operation time, the surgeons and patients also have less radiation exposure, which was essential for both patient's and surgeon's health. The current study showed that the excellent and good rate of functional outcomes (Majeed scores) was 88.9% and 87.5% in group 1 and group 2, respectively. There was no significant difference between group 1 and group 2 (*p* > 0.05). These functional outcomes certified that patients with unstable LC-1 pelvic fracture would get benefits from surgical stabilization [15]. The functional outcomes seemed better than the nonoperation, which was performed by Gaski et al. in the complete sacral fracture with displacement less than 1 cm [5].

The current study found that, in group 1, after the anatomic reduction and internal fixation of the anterior ring, the displacement of the posterior ring was also reduced, and there was no significant difference in the radiographic radiologic outcomes between this two groups (*p* > 0.05). Meanwhile, there was also no significant difference between the fixation patterns of the anterior ring (*p* > 0.05). The biomechanical study indicated that the retrograde screw could provide the comparable stability to reconstruction plate [31]; meanwhile, the latest comparative study showed that the modified pedicle screw-rod fixation (infix) and anterior external fixation could provide similar satisfactory clinical outcomes for anterior pelvic ring fracture [32]. Therefore, the selection of the implant for anterior ring fixation did not affect the outcomes in lc-1 pelvic fractures. The final follow-up of this group (group 1) showed that all sacral fractures healed. We deduced that after the anatomic reduction of the anterior ring, the posterior ring restored to the correct position, and the tension of the posterior ligament complex was reduced, which was beneficial to the healing of the posterior ring injury. Usually, the bilateral pubic ramus fractures were greater unstable than the unilateral pubic ramus fracture; however, there were no significant differences in functional outcomes after the ORIF(open reduction and internal fixation) of the anterior pelvic ring (*p* > 0.05). We believe that, with the anterior ring fixed, the stability of the whole pelvic ring was significantly increased. The results have been approved by the finite element analysis [33].

The current study also found that after stable fixation, in the early stage, there was a significant difference of weight-bearing between these two groups (*p* < 0.05). It seemed to be that the patients were more aggressive in weight-bearing in group 2, which might be attributed to pain relief.

The current study also found that the complications were no significant difference between group 1 and group2 (*p* > 0.05). The most frequent complication was VTE; 20.6% of the patients developed VTE (14/68, six silent DVT in group 1, six silent DVT and one nonfatal PE in group 2), which may be attributed to the regularly VTE screening preoperatively and postoperatively in our hospital. Although the thrombosis prophylaxis was routinely prescribed to the patients, Kim et al. reported a similar incidence of DVT (20%) [34]. However, in Kim et al.'s study, the rate of clinically significant VTE was much higher than the current study. In group 1, one patient got INFIX-related LFCN injury, which gradually recovered after the removal of the implant 3 months later. In group 2, one patient got postoperative L5 nerve root injury, with some residual weakness of extensor hallucis longus, which may due to the iatrogenic injury. Surgical exploration and decompression were offered, but the patient refused; The preoperative L5 injury gradually recovered in both groups. In group 2, one patient got pedicle screw-related skin irritation; the irritative symptoms recovered gradually after the implant was removed.

## 5. Limitations

There are some limitations to the current study. First, as a retrospective control study, the patients were not randomly divided into two groups. Second, the current study did not compare the outcomes with the incomplete displacement of the sacral fracture of LC-1 pelvic fractures, which may potentially expand the surgical indication. Third, although the final functional outcomes seem good through solitary anterior fixation, this fixation needs to be verified by the biomechanical study.

## 6. Conclusion

LC-1 PFCSFs can get benefits from ORIF; the treatment algorithm should be differently made following the degree of the sacral fractures displacement. Less than 1 cm sacral fracture displacement may get good functional outcomes from solitary anterior fixation. However, for the sacral fractures displacement greater or equal to 1 cm, both the anterior and posterior pelvic ring should be surgical stabilization.

## Figures and Tables

**Table 1:** Demographic, injury data, and surgical data of patients.

	Non-operative of posterior pelvic (group I, *n* = 36)	Operative of posterior pelvic (group II, *n* = 32)	*p* level
Age (mean ± SD, years)	38.1 ± 13.5	40.8 ± 15.5	0.447
Sex (male : female)			0.330
M	19	21	
F	17	11	
Mechanism of accident (*n*, %)			0.033
Crush by heavy	6 (16.7%)	2 (6.3%)	
MVA	13 (36.1%)	20 (62.5%)	
Fall from height ≥ 3 m	8 (22.2%)	1 (3.1%)	
Fall from height < 3 m	9 (25%)	9 (28.1%)	
ISS			0.520
<16	23	18	
≥16	13	14	
Anterior ring injury pattern			0.345
Ipsilateral	18 (50%)	20 (62.5%)	
Contralateral	7 (19.4%)	7 (21.9%)	
Bilateral	11 (30.6%)	5 (15.6%)	
Associated injury			0.796
None	20	13	
Head	2	3	
Thorax	3	4	
Abdomen	3	3	
Upper extremity	4	2	
Lower extremity	3	5	
Multi-injury	1	2	

Tips: MVA: motor vehicle accident; ISS: injury serious score.

**Table 2:** The comparison of surgical factors, radiological outcomes, and functional outcomes, between group 1 and group 2.

	Non-operative of posterior pelvic (group 1, *n* = 36)	Operative of posterior pelvic (group 2, *n* = 32)	*p* level
Fixation of anterior -ring			0.14
Plate	24	22	
Cannulated screw	6	9	
Infix	5	0	
Exfix	1	1	
Fixation of posterior -ring			—
Plate	NA	9 (28.1%)	
Cannulated screw	NA	16 (50%)	
Rod with pedicle screws	NA	7 (21.9%)	
Operation time (min, from incision to closure)	117.6 ± 46.0	158.9 ± 28.1	*p* < 0.01
Blood loss (ml)	187.5 ± 133.0	264.1 ± 158.2	0.034
Fluoroscopy time (seconds)	12.58 ± 3.7	74.28 ± 18.8	*p* < 0.01
Pain Preoperation	6.47 ± 1.61^∗^	7.47 ± 1.54^#^	*p* < 0.01^∗^ *p* < 0.01^#^
Postoperation	3.05 ± 0.98^∗^	4.06 ± 1.32^#^	
Early weight-bearing status			*p* < 0.01
Nonweight-bearing	9	7	
Partial weight-bearing	27	3	
Full weight-bearing	0	22	
Radiographic grades			
(Tornetta and Matta radiographic grading)			0.948
Excellent	29 (80.6%)	25 (78.2%)	
Good	4 (11.1%)	3 (9.4%)	
Fair	3 (5.5%)	3 (6.2%)	
Poor	0 (0%)	1 (3.1%)	
Function outcomes (Majeed scores grading)			0.693
Excellent	23 (63.9%)	16 (50.0%)	
Good	9 (25%)	12 (37.5%)	
Fair	3 (8.3%)	3 (9.3%)	
Poor	1 (2.8%)	1 (3.1%)	

Tips: ^∗^compare the preoperation with the postoperation in group 1. ^#^Compare the preoperation with the postoperation in group 2. NA: not available.

**Table 3:** The comparison of complications between group 1 and group 2.

Complications	Nonoperative of posterior pelvic (group 1, *n* = 36)	Operative of posterior pelvic (group 2, *n* = 32)	*p* level
			0.688
Nerve root injury	1 (preoperative)	3(2 patients preoperative, 1 patient postoperative)	
VTE	6 (DVT)	8 (7 DVT, 1 nonfatal PE)	
Implant-related	1 LFCN injury (recovered) 1 asymptomatic screw loosening and delayed union of the pubic rim	1 implant irritation	

VTE: venous thromboembolism; DVT: deep vein thrombosis; PE: pulmonary embolism; LFCN: lateral femoral cutaneous nerve.

## Data Availability

This article only includes summarized data from this study. Datasets are available from the corresponding author on reasonable request.

## Abbreviations

PICSF: Pelvic injuries with complete sacral fracture
ORIF: Open reduction and internal fixation
CT: Computerized tomography
MUA: Manipulation under anesthesia
LMWH: Low molecular weight heparin
VTE: Venous thromboembolism
DVT: Deep vein thrombosis
PE: Pulmonary embolism
LFCN: Lateral femoral cutaneous nerve
ISS: Injury severity score
VAS: Visual analog score.
